# A contemporary revision of the undergraduate oral and maxillofacial pathology syllabus and best practice recommendations

**DOI:** 10.1038/s41415-026-9948-4

**Published:** 2026-09-25

**Authors:** Daniel J. Brierley, Krishna Suchak, Anne Chambers, Oluyori Adegun, Adam V. Jones, Sharon J. White

**Affiliations:** 504115201236184204834 The School of Clinical Dentistry, Faculty of Health, The University of Sheffield, Sheffield, United Kingdom; 615135076160932474132 Barts Health National Health Service Trust and Institute of Dentistry, Queen Mary University, London, United Kingdom; 353856167919898912763 Barts Health National Health Service Trust and Institute of Dentistry, Queen Mary University, London, UK; Newcastle Upon Tyne Hospitals NHS Foundation Trust, Newcastle University, Newcastle, United Kingdom; 011280723692454978473 Oral and Maxillofacial/Head and Neck Pathology Unit, Department of Cellular Pathology, University College London Hospitals NHS Foundation Trust, London, United Kingdom; 998565907494249654158 Cellular Pathology and University Dental Hospital, Cardiff and Vale University Health Board, Cardiff, United Kingdom; 767983798872152819619 School of Dentistry, Faculty of Health, University of Dundee, Dundee, United Kingdom

## Abstract

This paper presents a fully revised undergraduate oral and maxillofacial pathology syllabus for dental schools in the UK, updating the 2004 British Society for Oral and Maxillofacial Pathology (BSOMP) minimum curriculum paper to reflect advances in diagnostic science, new disease entities, regulatory changes, and contemporary pedagogical practice. Developed by a BSOMP working group of consultant oral and maxillofacial pathologists, the updated syllabus incorporates current General Dental Council *Safe practitioner* requirements. The paper outlines the rationale for revision, describes the methodology, and provides recommendations for contemporary delivery including interdisciplinary integration, digital pathology and case-based learning.

**Supplementary Information:**

Zusatzmaterial online: Zu diesem Beitrag sind unter https://doi.org/10.1038/s41415-026-9948-4 für autorisierte Leser zusätzliche Dateien abrufbar.

## Key points

- A fully revised undergraduate oral and maxillofacial pathology syllabus, reflecting advances in disease knowledge, diagnostic science, and regulatory requirements.
- Recommendations include case-based learning, virtual microscopy and interdisciplinary integration to enhance clinical reasoning, diagnostic confidence, and engagement in dental students.
- With alignment to General Dental Council *Safe practitioner* outcomes, this update ensures graduates gain the foundational pathology knowledge needed for safe practice.

**Supplementary Information:**

Zusatzmaterial online: Zu diesem Beitrag sind unter https://doi.org/10.1038/s41415-026-9948-4 für autorisierte Leser zusätzliche Dateien abrufbar.

## Introduction

In 2004, the British Society for Oral and Maxillofacial Pathology (BSOMP) published a national minimum curriculum that supported UK dental schools in delivering essential knowledge in oral and maxillofacial pathology (OMFP).^1^ Since that time, the landscape of dental education has changed substantially. Newly characterised diseases such as medication-related osteonecrosis of the jaws (MRONJ) and human papillomavirus (HPV)-associated oropharyngeal carcinoma are now well recognised, and diagnostic practice has evolved with the development of immunohistochemistry and molecular techniques leading to more refined diagnoses and potential treatment options. In addition, both national regulatory frameworks and General Dental Council (GDC) learning outcomes have changed. These developments require an updated syllabus that reflects contemporary practice and supports the production of safe, competent dental graduates.

## Rationale for updating the curriculum

Major drivers for revision included:

- Evolving disease patterns – including MRONJ, HPV-driven malignancies and other relevant pathologies
- Advances in diagnostic science – notably immunohistochemistry and molecular testing
- Competency-based education – alignment with GDC *Safe practitioner* learning outcomes
- Integration with clinical disciplines – oral medicine, dental and maxillofacial radiology, oral surgery, and restorative dentistry
- Digital pedagogy and virtual microscopy – now central to pathology teaching
- Curricular time pressures – requiring a clearer identification of essential core content that should be delivered by subject specialists.

While learning outcomes are provided by the GDC, a detailed syllabus is not offered, necessitating the development of a syllabus that can inform OMFP teaching in undergraduate dental education. Moreover, no clear delineation is made of the interdisciplinary nature between OMFP and related specialties – such as oral medicine, microbiology, dental and maxillofacial radiology and oral surgery – which can hinder identification of specific oral pathology topics. Those GDC *Safe practitioner* learning outcomes and behaviours^2^ most relevant to OMFP, or at least having an oral pathology component, are shown in Table 1. This paper should be read in conjunction with the original 2004 publication,^1^ which offers comprehensive background and explanatory detail that is relevant – but not reiterated – in this updated syllabus.

**Table 1:** List of GDC Learning outcomes and behaviours from *The safe practitioner* relevant to OMFP teaching (note: some learning outcomes may be wholly relevant to OMFP teaching while others share crossover with other specialties)

**GDC learning outcome**	**Description**
C 1.1	Explain the aetiology, pathogenesis and epidemiological trends of oral and dental disease and their application to patient management
C 1.2	Describe and identify the clinical presentations of oral and dental diseases and explain the principles underpinning their diagnosis, prevention, and treatment
C 1.3	Explain the variance in disease presentation across diverse cultural and social groups, and those with protected characteristics, and how this impacts diagnosis, prevention, and treatment
C 1.4	Describe and identify general and systemic diseases and psychological conditions, and their relevance to oral health and impact on clinical treatment, patient compliance, self-care, and outcomes
C 1.13	Evaluate the health risks of prescribed, non-prescribed and recreational drug use and misuse on oral and general health and how to provide appropriate advice and support including signposting or referral
C 1.30	Describe the aetiology and pathogenesis of diseases of the oral and maxillofacial complex
C 1.31	Identify all stages of malignancy, the aetiology and development of tumours and the importance of early referral for investigation and biopsy
C 1.37	Identify and explain the principles of when and how to refer patients for specialist treatment and apply to practice
C 2.1.4	Appropriately prescribe and/or interpret the findings of clinical and laboratory investigations
C 2.1.5	Undertake relevant special investigations and diagnostic procedures, including radiography
C 2.1.7	Synthesise the full results of the patient's assessment and make clinical judgements taking into account patient compliance, values, cultural identity, and self-care
C 2.1.8	Formulate a differential diagnosis or diagnoses and from there a definitive diagnosis
C 2.1.9	Formulate a personalised treatment plan, synthesising patient assessment, diagnostic data, prognosis, and shared decision making
C 2.2.4	Safely and appropriately prescribe and administer medicines and therapeutic agents
C 2.4.3	Diagnose and manage acute dento-alveolar and mucosal infection
I 1.6	Use appropriate methods to provide accurate, clear and comprehensive information when referring patients to other dental and healthcare professionals
I 1.7	Communicate appropriately and effectively in professional discussions and transactions
I (B)6	Where appropriate manage and refer/delegate work according to the scope of practice of members of the dental team, in line with competence and professional practice
P (B)8	Act in accordance with current best practice guidelines
GDC, General Dental Council; OMFP, oral and maxillofacial pathology.	

## Methods

The syllabus revision was undertaken by a BSOMP working group comprising OMFP consultants across the UK, overseen by the Teacher's Group Chair. The invitation to contribute to the syllabus was shared with all BSOMP members – the final group consisted of six experienced pathologists who teach OMFP (and often related fields) in their respective dental school (six separate institutions). The group reviewed the original 2004 curriculum line by line, compared historical content with contemporary clinical practice and disease epidemiology, and mapped expected knowledge domains to the GDC *Safe practitioner* outcomes.

Through structured discussion, the group reached consensus on additions, removals, and revisions. The updated syllabus was shared with all BSOMP members over email with an opportunity for members to offer anonymous feedback. Three comments were received which led to minor amendments only. An updated list of entities, including MRONJ, HPV-associated oropharyngeal carcinoma, expanded soft-tissue and skin tumours, neck lumps and an updated list of salivary gland tumours, were incorporated accordingly. The updated syllabus topics can be seen in the online Supplementary Information.

In addition, the group discussed how they currently taught OMFP within their respective dental school. These discussions led to agreement on recommended best practices when teaching pathology to dental students.

## Discussion

Over the past two decades, significant shifts in medical, dental, and higher education have reshaped how pathology must be taught at undergraduate level.^3^^,^^4^^,^^5^^,^^6^^,^^7^ Multiple reports across medicine and dentistry describe a progressive reduction in dedicated pathology teaching time, driven by curriculum overcrowding and structural pressures within universities such as reductions in specialist academic staff.^8^ These changes have resulted in concerns regarding pathologists' ability to deliver essential foundational science content and the risk that students may graduate with weaker understanding of disease mechanisms, diminished confidence in interpreting diagnostic investigations, and reduced preparedness for clinical decision-making. This may in turn reduce willingness to pursue the specialty at postgraduate level. This updated paper therefore responds to a recognised need to reinforce pathology's central role in safe, evidence-based dental practice. While an updated syllabus is presented here, the following discussion is important to consider for context and delivery of the syllabus.

### Pathology teaching in an era of reduced curricular time

Across UK medical and dental schools, pathology teaching has been consistently reported as becoming down-sized, fragmented, or dispersed across disciplines.^8^^,^^9^^,^^10^^,^^11^^,^^12^^,^^13^ Traditional lecture- and laboratory-based courses have been replaced by blended or systems-based learning models which, although pedagogically beneficial, risk unintentionally de-emphasising core pathological principles and perception of relevance, if pathology specialists are not embedded within curriculum design. Similar concerns have been raised in medical literature, where decreased exposure to pathology correlates with reduced student confidence in diagnostic reasoning, specimen handling, and interpretation of laboratory results.^14^ Dentistry shares these risks, especially given that early oral manifestations of systemic disease and head-and-neck malignancies rely on a strong grounding in pathological mechanisms for timely recognition.

The updated syllabus therefore acts not only as a content guide but also as a strategic safeguard-helping dental schools protect essential foundational science when curricular hours are compressed.

### Advancements in diagnostic science and their implications for dental education

Since the original syllabus was published in 2004, diagnostic pathology has undergone profound transformation. Key developments include:

- Digital pathology and whole-slide imaging, now used for routine diagnostics and increasingly central in teaching
- Immunohistochemistry becoming standard for characterising many tumours including salivary gland tumours
- Molecular diagnostics, including detection of HPV in oropharyngeal cancer, genetic profiling of fibro-osseous lesions, and mutation-based classification of odontogenic tumours. Some of these have potential gene specific targets which may lead to modified interventions
- Artificial intelligence-assisted image analysis, which is prominent in pathology research and is likely to influence future diagnostic pathways.

As these advancements evolve, those teaching pathology to students will also need to evolve to meet the ever-changing diagnostic landscape. While dentists are not expected to perform or interpret complex tests, they must appreciate the principles, indications, and limitations of modern diagnostic tools, understand how pathologists reach a diagnosis, and have some form of appreciation of when advanced investigations are required. The updated syllabus reflects this by including reference to immunohistochemistry, molecular testing, and digital imaging – all of which were absent from the 2004 curriculum.

### The role of pathology in clinical decision-making

Despite pressures to reduce didactic basic science teaching, pathology remains a core explanatory science of dentistry. Competent dental graduates must:

- Recognise normal and abnormal tissues
- Construct appropriate differential diagnoses
- Identify potentially malignant and malignant lesions
- Interpret pathology reports
- Appreciate when, and why, specialist referral is required.

### Modern approaches to teaching pathology

Given the variability across UK dental schools in teaching time, staff availability, and integration models, a contemporary BSOMP syllabus provides consistency and clarity. It helps ensure that all graduates, regardless of institution, achieve the essential learning outcomes required by the GDC and are prepared for safe clinical practice. A growing literature supports innovative educational strategies in pathology:

- Virtual microscopy has been shown to improve learner engagement, support asynchronous learning, reduce equipment inequality, and enhance accessibility for students with disabilities.^15^^,^^16^^,^^17^^,^^18^ As such, virtual microscopy is strongly recommended over traditional slides and a microscope when teaching pathology to students
- Case-based learning (CBL) and problem-based learning (PBL) can foster deeper conceptual understanding, integrating pathology with clinical reasoning and diagnostic decision-making, thereby increasing students' perception of relevance and reducing compartmentalised learning. These methods of teaching are also recommended, which can be supported by traditional lecture content, perhaps using a flipped learning approach
- Digital platforms, social media and gamification allow dental students to practise diagnostic interpretation in a controlled, feedback-rich environment.^19^ Some dental schools have supported their students with learning pathology through YouTube with considerable success.^20^ These methods can consolidate more traditional teaching methods and appeal to current students who value these approaches compared to older generations^21^
- Interdisciplinary teaching, bringing together OMFP, dental and maxillofacial radiology, oral medicine, and oral surgery, reflects real-world workflow in the diagnosis of oral disease and head and neck cancer MDTs improving students' diagnostic confidence and understanding of a patient's journey through cancer treatment. Teaching pathology using an integrated approach helps students see the role of pathology in patient care and should be encouraged.

Case-based learning lends itself to small-group teaching (approximately 6–10 students) where pathology topics from the syllabus list can be explored in detail. For example, in one of the author's institutions, students are assigned a particular scenario to work through by a staff member at the start of the session. Each student is given around 30 minutes to examine a clinical description of the patient's presenting complaint, alongside a clinical or radiological image and virtual microscopy slide. After 30 minutes, each student presents their case to the group, in turn, encouraging peer learning. The staff member acts as the facilitator of the discussions to aid and check student understanding. The entire session lasts about 2–3 hours. For example, a relevant clinical scenario might be on the topic of a non-healing ulcer (Table 2).

**Table 2:** Example areas of discussion for a clinical scenario alongside the relevant GDC learning outcomes and behaviours from *The Safe Practitioner*^2^

**Clinical scenario: non-healing ulcer/oral cancer**	
**Area of focus**	**GDC learning outcome**
1) The student describes the salient features from the clinical image or radiograph and suggests the most pertinent questions to ask the patient	C 1.2, C 1.3, C 1.31
2) A differential diagnosis based on the information provided	C 1.2, C 1.3, C 2.1.8
3) The most relevant referral pathway and need for biopsy	C 1.31, C 1.37, I 1.6, P (B)8
4) Any other special investigations or imaging relevant to the case	C 1.2, C 1.31, C 2.1.4, C 2.1.5
5) A review of the histology (digital images) from the biopsy	C 1.2, C 1.30, C 2.1.4
6) The nature of the disease (categorisation, aetiology, pathogenesis, etc.)	C 1.1, C 1.3, C1.30, C 1.31
7) The management of the case in both primary and secondary care	C 1.2, C 1.3, C 1.4, C. 1.13, C 2.1.7, C 2.1.9, C 2.2.4, I 1.7, I (B)6, P (B)8
8) The prognosis and long-term effects of the disease and its management	C 1.2, C 1.3, C1.4, C 1.13, C 2.1.9
GDC, General Dental Council.	

In this format, there would be multiple opportunities to link pathology teaching to other dental specialties. For example, under focus area three in Table 2, the student could be asked about the biopsy technique (oral surgery) and for focus area four, dental and maxillofacial radiology teaching would be relevant, with example head and neck imaging included for the student to discuss.

This case-based, small-group approach to pathology teaching provides multiple pedagogical benefits. By integrating virtual microscopy, clinical scenarios, and interdisciplinary input, students not only acquire the essential factual knowledge but also develop critical clinical reasoning skills, diagnostic confidence, and an appreciation of the patient journey. Linking pathology teaching to other dental specialties allows students to see the relevance of the subject in real-world clinical practice, reducing compartmentalised learning and fostering a more holistic understanding of oral healthcare. Furthermore, the active learning format encourages peer discussion and self-directed inquiry, giving staff opportunities to identify and address knowledge gaps in real-time.

To optimise pathology teaching across dental schools, it is recommended that curricula adopt a structured, yet flexible, approach that combines: virtual microscopy for accessibility and engagement; CBL or PBL to enhance conceptual understanding; digital tools (such as YouTube and gamification) to reinforce learning; and interdisciplinary sessions to reflect clinical realities. Small-group, case-focused sessions, supported by experienced facilitators, should form the core of this model, supplemented by lectures or flipped classroom resources to consolidate foundational knowledge. By standardising the learning outcomes through a contemporary BSOMP syllabus while allowing flexibility in teaching methods, dental schools can ensure graduates are well-prepared for safe, competent, and confident clinical practice.

Examples of other OMFP topics which could be delivered in this way are shown in Table 3.

**Table 3:** Example scenarios which can be used to deliver undergraduate pathology teaching alongside relevant topic

**Scenario**	**Examples of relevant OMFP topics**
Swelling on the gingiva	Fibroepithelial polyp; pyogenic granuloma; giant cell granuloma
White patch on the tongue	Frictional keratosis; dysplasia; candidiasis
Pigmented lesion in the palate	Melanotic macule; malignant melanoma; drug-induced pigmentation; amalgam tattoo
Swelling in the neck	Reactive lymph nodes; metastasis; lymphoma; developmental cysts
Sores in the mouth	Recurrent aphthous stomatitis; herpes simplex infection; syphilis; vesiculobullous conditions; lichen planus
A lump on the tongue	Granular cell tumour; salivary gland tumours; lipoma; neurofibroma
Swollen lips	OFG; Crohn's, sarcoidosis; tuberculosis; lip fillers
Swelling of the palate	Salivary gland tumours; mucous retention cysts
Swelling in the parotid gland	Salivary gland tumours; Sjögren's; sialosis
Radiolucency in the jaw	Odontogenic and non-odontogenic cysts; odontogenic tumours
Discoloured teeth	Amelogenesis imperfecta; dentinogenesis imperfecta
Radiopacity in the maxilla/mandible	Cemento-ossifying fibroma; fibrous dysplasia; cemento-osseous dysplasia; odontome
OFG, orofacial granulomatosis; OMFP, oral and maxillofacial pathology.	

## Conclusion

This updated syllabus modernises the BSOMP 2004 minimum curriculum, aligning it with contemporary disease knowledge and regulatory requirements. It defines the essential topics necessary for the safe-beginner dentist and provides dental schools with guidance on the pathological concepts that could be delivered through case-based learning, integrated clinical teaching, and digital tools. By standardising learning outcomes, the syllabus ensures consistency across institutions while retaining flexibility in teaching approaches, allowing schools to adapt to their staffing, resources, and local expertise. The integration of virtual microscopy, interdisciplinary sessions, and active learning strategies supports deeper understanding, clinical reasoning, and appreciation of the patient journey, ultimately preparing graduates for safe and competent practice. Adoption of this modernised curriculum promotes not only knowledge acquisition but also engagement, confidence, and readiness for real-world dental practice, addressing both educational variability and evolving professional expectations.

## Supplementary Information

Supplementary Information (PDF 151KB)
