# Supplementary material for: A contemporary revision of the undergraduate oral and maxillofacial pathology syllabus and best practice recommendations
Source: Br Dent J. 2026 Sep 25;241(6):411–5. doi: 10.1038/s41415-026-9948-4 (PMC13614893; doi:10.1038/s41415-026-9948-4)
Supplement: Supplementary file 1 [file 41415_2026_9948_MOESM1_ESM.pdf]

Appendix 1: Updated Oral and Maxillofacial Pathology Syllabus for undergraduate dental education

Category	Content
General topics	The processes of differential diagnosis, principles of history taking, examination, selection and interpretation of investigations for oral and head and neck disease.<br>Detailed knowledge of biopsy procedures for the mouth. Principles of biopsy techniques suitable for deep structures of the head and neck.<br>Specimen handling and interpretation of histology reports and other investigation results, including an awareness of immunohistochemistry and molecular testing in diagnosis.<br>An appreciation of the relative incidence of lesions and conditions.<br>Correct definition, use and spelling of medical and pathological terms.
Disorders of the teeth	Developmental disorders of the teeth:<br>-Missing and supernumerary teeth, morphological variations.<br>-Amelogenesis imperfecta, osteogenesis imperfecta, dentinogenesis imperfecta and dentine dysplasia.<br>-Chronological hypoplasia and fluorosis.<br>-Resorption and hypercementosis.<br>-Delayed eruption and accelerated tooth loss.
Dental caries	Enamel, dentine and root caries including caries in deciduous teeth, aetiology, pathogenesis and structural changes in enamel, dentine and cementum.
Pulpitis and pulpal reactions to damage	Apical periodontitis and the sequelae of pulp necrosis or pulp removal:<br>-Periapical granuloma.<br>-Radicular cyst.<br>-Dento-alveolar abscess and spread of infection.<br>Tooth wear and the processes of attrition, abrasion and erosion.
Disorders of the tooth supporting structures	Plaque-related gingivitis, periodontitis and their variants including classification, aetiology, pathogenesis and structural changes.<br>Acute ulcerative gingivitis.<br>Periodontal abscess.<br>Pericoronitis.<br>Causes of localised and generalised gingival enlargement.
Disorders of the oral mucosa	Genetic conditions:<br>-White sponge naevus.<br>Hyperplasias:<br>-Fibrous epulis.

Category	Content
Disorders of the oral mucosa (cont)	-Fibro-epithelial hyperplasia.<br>-Pyogenic granuloma.<br>-Peripheral giant cell granuloma.<br>-Squamous papilloma.<br>Soft tissue tumours:<br>-Lipoma<br>-Haemangioma<br>-Lymphangioma<br>-Neurofibroma<br>-Schwannoma<br>-Traumatic neuroma<br>Inflammatory disorders:<br>-Traumatic injury including, mechanical, chemical exposure and thermal.<br>-Radiation exposure and the effects of radiation on tissues.<br>Local and systemic infections:<br>Bacterial:<br>-Infections of dental origin<br>-Abscess<br>-Cellulitis<br>-Tuberculosis<br>-Actinomycosis<br>-Syphilis<br>Viral:<br>-Primary and recurrent herpes simplex infection<br>-Herpes zoster infection<br>-Herpangina and hand foot and mouth disease<br>-Epstein–Barr virus infection and its sequelae<br>Fungal:<br>-Candidosis<br>Local and systemic immunopathogenic conditions:<br>-Recurrent oral ulceration<br>-Behcet's disease<br>-Lichen planus and lichenoid reactions<br>-Lupus erythematosus

Category	Content
Disorders of the oral mucosa (cont)	Vesiculobullous conditions:<br>-Pemphigus<br>-Pemphigoid<br>-Erythema multiforme<br>-Angina bullosa haemorrhagica<br>HIV infection and its sequelae<br>Oral complications of cancer therapy and transplantation:<br>-Chemotherapy-induced oral mucositis<br>-Immunotherapy-related oral immune-related adverse events (irAEs)<br>-Oral manifestations of chronic graft-versus-host disease (GVHD)<br>Local and systemic granulomatous conditions NOS:<br>-Crohn's disease<br>-Orofacial granulomatosis<br>-Foreign body reactions<br>-Sarcoidosis<br>Keratoses and white lesions NOS:<br>-Frictional and reactive keratoses<br>-Idiopathic lesions, 'leukoplakia'<br>Oral mucosal pigmentation:<br>-Racial pigmentation<br>-Melanotic macules<br>-Melanocytic naevi<br>-Peutz–Jegher disease<br>-Addison's disease<br>-Amalgam tattoo<br>-Melanoma<br>Oral potentially malignant disorders (OMPD):<br>-Concept of OPMD<br>-OPMDs and conditions<br>-Epithelial dysplasia<br>-Factors potentiating malignant change<br>Oral squamous cell carcinoma and oropharyngeal HPV associated squamous cell carcinoma:<br>-Epidemiology

Category	Content
Disorders of the oral mucosa (cont)	-Aetiology<br>-Histopathology<br>-Patterns of spread<br>-Factors affecting prognosis and management<br>-Prevention<br>Other malignancy:<br>-Lymphoma/leukaemia<br>-Basal cell carcinoma<br>-Squamous cell carcinoma of skin<br>-Malignant melanoma of skin
Disorders of facial bones and jaws	Developmental conditions:<br>-Tori<br>-Cleft palate<br>-Cleidocranial dysplasia<br>Inflammatory disorders:<br>-Osteomyelitis of the jaws and osteoradionecrosis<br>-Healing fracture and tooth socket<br>-Dry socket<br>-Medication related osteoradionecrosis of the jaws (MRONJ)<br>Cystic and cyst-like lesions:<br>-Classification of jaw cysts<br>-Odontogenic cysts<br>-Radicular cyst<br>-Inflammatory collateral (paradental) cyst<br>-Dentigerous cyst<br>-Odontogenic keratocyst and basal cell naevus syndrome (Gorlin syndrome)<br>Non-odontogenic cysts:<br>-Incisive canal cyst<br>-Solitary bone cyst<br>-Aneurysmal bone cyst<br>-Stafne/idiopathic bone cavity<br>Soft tissue cysts:

Category	Content
Disorders of facial bones and jaws (cont.)	-Dermoid<br>-Branchial<br>-Thyroglossal<br>Odontogenic tumours:<br>-Principles of classification and the range of odontogenic tumours, specifically:<br>-Odontomes<br>-Ameloblastoma<br>-Cementoblastoma<br>-Adenomatoid odontogenic tumour<br>Giant cell and other lesions of bone:<br>-Central giant cell granuloma and hyperparathyroidism (brown tumour)<br>-Paget's disease<br>-Langerhans cell histiocytosis<br>Fibro-osseous lesions:<br>-Fibrous dysplasia<br>-Cemento-ossifying fibroma (ossifying fibroma)<br>-Cemento-osseous dysplasias (osseous dysplasias)<br>Disorders of the temporomandibular joint and masticatory apparatus:<br>-Myofascial pain dysfunction syndrome<br>Bone neoplasms:<br>Osteoma including Gardner's syndrome<br>Osteosarcoma<br>Metastatic neoplasms to the jaws
Salivary gland disease	Developmental disorders:<br>Mucocele:<br>-Mucous extravasation cyst<br>-Mucous retention cyst<br>Sialolithiasis, obstruction and chronic sialadenitis.<br>Infections:<br>-Mumps<br>-Acute and chronic bacterial sialadenitis<br>Disturbances of salivation:<br>-Causes of xerostomia

Category	Content
Salivary gland disease (cont.)	-Sjögren's syndrome<br>-Radiotherapy-induced salivary gland atrophy<br>-Ptyalism/sialorrhoea<br>Salivary neoplasms:<br>-Principles of classification and the range of salivary neoplasms:<br>-Pleomorphic adenoma<br>-Warthin tumour<br>-Canalicular adenoma<br>-Mucoepidermoid carcinoma<br>-Polymorphous adenocarcinoma<br>-Adenoid cystic carcinoma<br>-Carcinoma ex pleomorphic adenoma<br>-Acinic cell carcinoma
Neck lumps	An awareness of the range of conditions that can present as a lump in the neck:<br>-Metastatic disease<br>-Reactive lymphadenopathy and lymphomas<br>-Relevant soft tissue tumours<br>-Neck cysts
